# Case Report: Novel truncating PPM1D variant in a dichorionic diamniotic (DCDA) twin with Jansen-de Vries syndrome. an updated perspective

**DOI:** 10.3389/fgene.2025.1601752

**Published:** 2025-06-24

**Authors:** Francisco Javier Merida De la Torre, Javier Porta Pelayo, Inmaculada Ortiz-Martín

**Affiliations:** ^1^ Genetics Laboratory, Hospital Regional Universitario, Málaga, Spain; ^2^ Genomics, Genologica, Málaga, Spain

**Keywords:** Jansen-de Vries syndrome, PPMD1, developmental delay, intellectual disability, case report

## Abstract

**Introduction:**

Jansen-de Vries syndrome (JDVS) is a rare autosomal dominant neurodevelopmental disorder caused by truncating variants in exons 5 and 6 of the *PPM1D* gene. Its diagnosis is often delayed due to symptom overlap with more common conditions such as autism spectrum disorder (ASD) and attention-deficit/hyperactivity disorder (ADHD). This case report highlights the unique presentation of JDVS in one of a pair of dichorionic diamniotic (DCDA) twin brothers, both with ASD/ADHD, underscoring the diagnostic value of genetic testing.

**Case presentation:**

A 6-year-old boy presented with delayed language development, learning difficulties, behavioral issues, restrictive eating, and impaired autonomy. His twin brother, although also diagnosed with ASD/ADHD, exhibited milder symptoms. Trio-whole-exome sequencing revealed a *de novo* frameshift mutation (c.1411_1412del) in *PPM1D* in the proband, classified as pathogenic. The brother had no such variant.

**Interventions and outcomes:**

The proband received multidisciplinary interventions including behavioral therapy and speech support. Follow-up showed improvements in language, sleep, and academic performance, though behavioral and sphincter issues persist. The twin without the mutation was discharged from mental health services, while his brother remains under annual review.

**Conclusion:**

This case emphasizes the expanding phenotypic spectrum of JDVS and illustrates the diagnostic value of trio-WES in neurodevelopmental disorders with overlapping features. It also highlights the potential for discordant phenotypic expression in twins and the need for individualized diagnostic assessment.

## 1 Introduction

JDVS (OMIM 617450, MONDO 0044318) was first described by Jansen et al. (2017) as a heterogeneous neurodevelopmental disorder characterised by diverse intellectual disability severity, hypotonia, wide gait, sensitivity to sound and behavioural problems. Affected individuals also exhibit dysmorphic skeletal and facial features, including short stature, small hands and feet, hyperlordosis, broad forehead, low-set, posteriorly rotated ears, upturned nose, thin upper lip and broad mouth. Other common symptoms include fever and vomiting episodes, high pain threshold, feeding difficulties and vision problems. Jansen and colleagues identified 14 truncating germline mutations in the last two exons (exons 5 and 6) of the PPM1D gene in the same number of unrelated patients as possible causes of this newly described disorder.

The PPM1D gene (CCID:007703), which encodes a member of the PP2C family of Ser/Thr protein phosphatases, has been shown to act as a negative regulator of cellular stress response pathways. This phosphatase plays a role in the feedback regulation of the p38-p53 signalling pathway, contributing to growth inhibition and suppression of stress-induced apoptosis. It is induced in a p53-dependent manner in response to various environmental stresses and inhibits the activity of p38 MAP kinase (MAPK/p38), which in turn reduces p53 phosphorylation and suppresses p53-mediated transcription and apoptosis.

The truncating mutations of the PPM1D gene described thus far are likely to evade nonsense-mediated mRNA decay, resulting in stable truncated transcripts. While these alterations do not affect p53 activation, they cause a growth disadvantage in cells, suggesting an impact on the stress-response pathway. This research underscores the significance of cell-cycle checkpoint genes in the context of neurodevelopmental disorders (Jansen et al., 2017).

**FIGURE 1:**
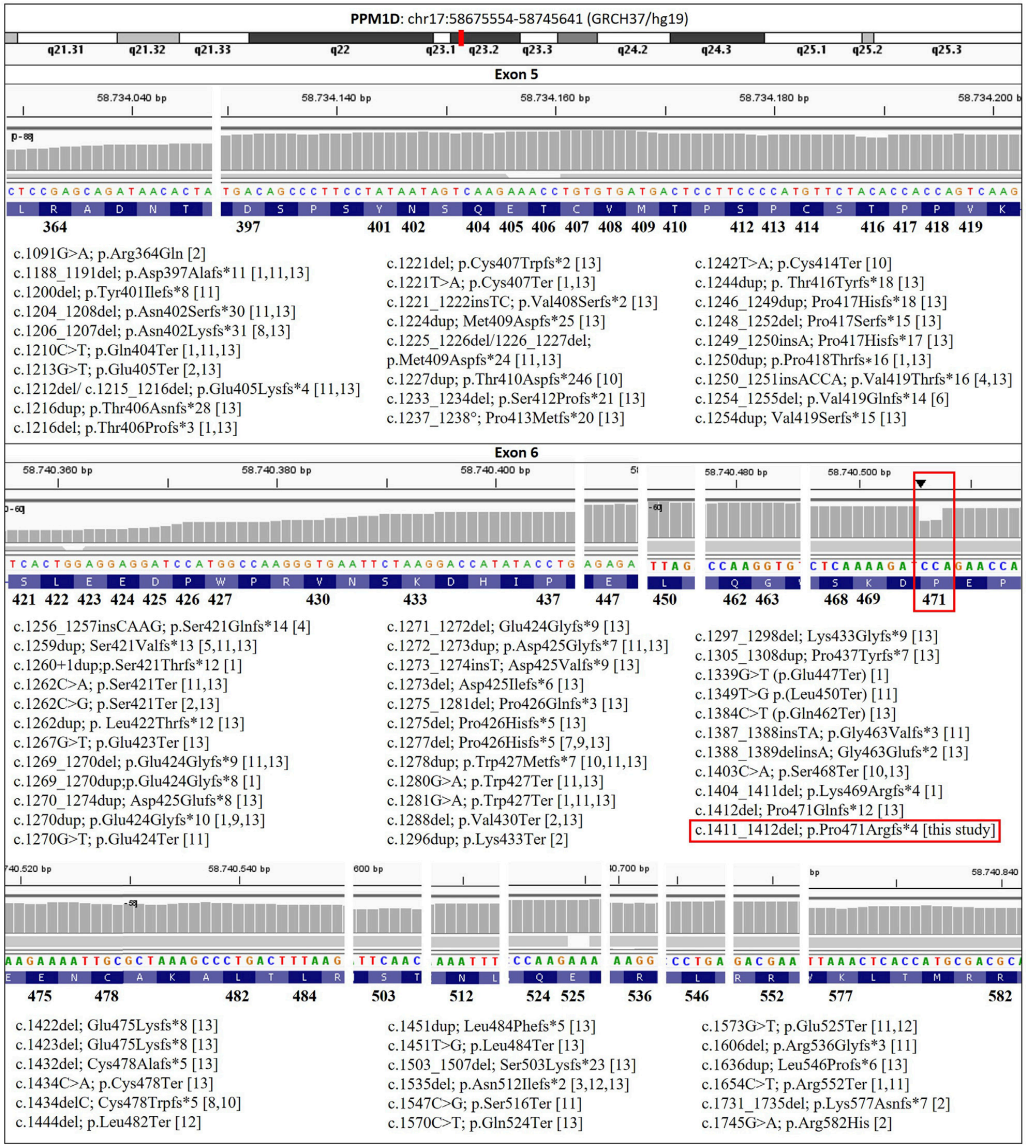
PPM1D variants associated with Jansen-de Vries syndrome reported in the literature, in this case (red square), and in the ClinVar database to date (https://www.ncbi.nlm.nih.gov/clinvar/). [1] Jansen et al., 2017; [2] Stessman et al., 2017; [3] Porrmann et al., 2019; [4] Kuroda et al., 2019; [5] Schmitz-Abe et al., 2019; [6] Li et al., 2020; [7] Tran Mau-Them et al., 2020; [8] Fernández-Mayoralas et al., 2021; [9] Tsai et al., 2022; [10] Tuiskula et al., 2023; [11] Wojcik et al., 2023; [12] Cunningham et al., 2024; [13] ClinVar (Landrum et al., 2018). Reference sequence NM_003620.4.

A review of the literature reveals that 27 frameshift, 16 nonsense, 1 splice site and 2 missense mutations have been reported in exons 5 and 6 of PPM1D (Jansen et al., 2017; Stessman et al., 2017; Porrmann et al., 2019; Kuroda et al., 2019; Schmitz-Abe et al., 2019; Li et al., 2020; Tran Mau-Them et al., 2020; Fernández-Mayoralas et al., 2021; Tsai et al., 2022; Tuiskula et al., 2023; Wojcik et al., 2023; Cunningham et al., 2024). A total of 57 truncating variants (43 frameshift and 14 nonsense) have been categorised as pathogenic or likely pathogenic in the ClinVar database (Landrum et al., 2018; Landrum et al., 2025). To our knowledge, 22 out of 43 frameshift and 3 out of 14 nonsense mutations have not been previously documented in the literature (Figure 1).

The present study details the case of six-year-old diamniotic and dichorionic twin brothers diagnosed with ASD and ADHD. One of the twins (hereafter referred to as “twin 1”or the “proband”) has a *de novo* frameshift mutation in the last exon of PPM1D, and, in contrast to his brother (hereafter referred to as “twin 2”), he exhibits delayed language acquisition, learning difficulties, behavioural problems characterised by aggressiveness, lack of autonomy, problems with sphincter control and restrictive feeding.

## 2 Case description

The patient was referred from the neuropediatric office of the Maternal and Child Hospital of Malaga due to delayed language acquisition and hyperactive behaviour, in addition to his diamniotic and dichorionic brother.

Table 1 provides a synopsis of the clinical characteristics of JDVS that have been documented in the literature, in addition to those exhibited by the proband. All variants identified to date are shown in Figure 1, including the one described in this study. More detailed information about the phenotype can be found in the Supplementary Material (Supplementary Table S1).

**TABLE 1:** Clinical features of JDVS patients.

General information	This study	Reported cases^a^
Sex	6 years	41 males, 21 females, 1 NK
Age	Male	6mo-62y
Development and behaviour		
Hypotonia	yes (infantile)	40/56 (71%)
Psychomotor developmental delay	yes	37/40 (93%)
Intellectual disability	yes	53/60 (88%)
Behavioral features	yes	40/59 (67%)
High threshold to pain	yes	25/45 (56%)
Growth		
SGA	yes	10/53 (19%)
Short stature	no	33/54 (61%)
Microcephaly	no	14/38 (37%)
Dysmorphic facial features		
Wide forehead	no	44/60 (73%)
Low-set posteriorly rotated ears	no	15/19 (79%)
Upturned nose	no	10/22 (45%)
Thin upper lip	yes	49/58 (84%)
Wide mouth	no	47/58 (81%)
Other	—	35/46 (76%)
Other dysmorphic features		
Small hands	no	38/54 (70%)
Brachydactyly	no	6/8 (75%)
Small feet	no	35/52 (67%)
Dysplastic nails	no	26/54 (48%)
Hyperlordosis	no	11/16 (69%)
Other	Clynodactily/flat feet	7/10 (70%)
Gastrointestinal symptoms		
Feeding difficulties	yes	15/20 (75%)
GER and/or vomiting	yes	34/54 (64%)
Constipation	yes	36/51 (71%)
Other features		
Cardiac condition	no	6/11 (55%)
Structural changes in brain MRI	no	12/18 (67%)
Ophtalmological abnormalities	no	32/45 (71%)
Other	Sphincter control difficulties	43/62 (69%)

^a^ Jansen et al., 2017; Porrmann et al., 2019; Kuroda et al., 2019; Li et al., 2020; Fernández-Mayoralas et al., 2021; Tsai et al., 2022; Tuiskula et al., 2023; Wojcik et al., 2023; Cunningham et al., 2024. SGA: small for gestational age; GER: gastroesophageal reflux; MRI: magnetic resonance imaging; NK: Not known. This table has been adapted from Tuiskula et al. (2023).

### 2.1 Clinical history

The diamniotic and dichorionic fraternal twins were referred to the genetics service of the Materno Infantil Hospital in Malaga, Spain, for diagnosis of global developmental delay. The *in vitro* fertilisation babies were born at 38 weeks gestation via caesarean section delivery. Two embryos had been implanted, which were the only viable ones. Their birth parameters were as follows: weight 2,290 g (P3), height 48.0 cm (P15), and head circumference 32.0 cm (P3) for twin 1, and weight 2,890 g (P15), height 48.0 cm (P15), and head circumference 33.0 cm (P15) for twin 2. A normal physical evaluation was conducted, with the exception of clinically insignificant achromic patches. Both siblings presented with a normal phenotype and well-preserved general condition, with adequate hydration, nutrition, and perfusion. Cranial shape was normal, and neck mobility was intact with no lymphadenopathy. The chest was unremarkable; cardiac examination revealed normal heart sounds without murmurs, and pulmonary auscultation showed good bilateral ventilation. The abdomen was soft, non-tender, with no palpable masses or organomegaly. Musculoskeletal assessment revealed normal muscle appearance and no joint inflammation. Neurologically, the patients were alert and conscious, interacted with their environment though mildly distracted, and exhibited normal tone, strength, and posture. Cranial nerves were intact; pupils were equal, round, and reactive to light, with normal convergence, conjugate gaze, and smooth pursuit. Deep tendon reflexes were within normal limits. Gait and balance were appropriate for their age. Twin 1 exhibited clinodactyly, a condition that was resolved through the administration of orthopaedic treatment. Twin 2 demonstrated a valgus foot posture, a consequence of an intrauterine postural alteration.

The subjects were both diagnosed with ADHD and ASD by the mental health service of the same hospital when they were 2 years old, respectively. Subsequent diagnostic procedures did not reveal any anomalies. Electroencephalogram results demonstrated normal baseline brain bioelectrical activity for age, excluding paroxysmal abnormalities of epileptiform character. Brain MRI scans revealed no significant pathological changes, and audiometry results were within normal limits. Despite the apparent commonalities in symptoms, significant disparities emerge in the physical and psychological manifestations of their neurodevelopmental disorder.

Twin 1 demonstrated delayed language acquisition and erratic behaviour at the age of 2 years and 11 months. Specifically, he demonstrated an inability to construct coherent sentences and his play was primarily symbolic in nature. Social interaction was found to be within normal parameters; however, the subject displayed a tendency to resist instructions and exhibited mannerisms. The assessment with the M-CHAT in 2020 showed twelve alert items. After the administration of the M-CHAT questionnaire, the patient was assessed using the ADOS-2 Module 1 questionnaire, obtaining a score of 8 points, which rules out the diagnosis of autism. No additional questionnaires were administered. At the age of four, the patient continued to demonstrate challenges with learning and speech. At the age of five, the patient continued to demonstrate challenges with language and social interaction, though there was a discernible enhancement in his vocabulary. The patient demonstrated a pronounced temperament, marked inflexibility, and recurrent tantrums. Notably, his behaviour included an age-inappropriate interest in sexual organs, including those of his parents. However, once he had become familiar with the environment, he demonstrated an improvement in his ability to tolerate interaction with children. There was a marked improvement in his sleep. He exhibited a tendency to monopolise his mother’s attention and completed kindergarten with challenges, subsequently being promoted to the next level. Persistent difficulties with stool control were noted, as was a high tolerance to pain. Presently, he continues to exhibit behavioural problems and lacks a social smile, occasionally manifesting outbursts of anger that escalate to aggression towards his mother. In the educational context, he encounters challenges in the acquisition of knowledge. The subject displays an absence of autonomy, in addition to issues with sphincter control and restrictive eating. He also shows a slower developmental progress compared to their sibling. Notably, he exhibits manipulative and defiant behavior, low frustration tolerance, and sexualized conduct, which have not been observed in the other sibling.

In contrast, twin 2 exhibited a general delay in cognitive development at the age of three, as evidenced by performance on standardised tests, in comparison to his school-aged peers. He has a tendency to be alone and does not socialise with his brother either. He is currently under the care of a mental health service that specialises in high-functioning autism and ADHD. The subject has demonstrated an innate capacity for spontaneous reading skills, and his progress in this domain is noteworthy.

**FIGURE 2:**
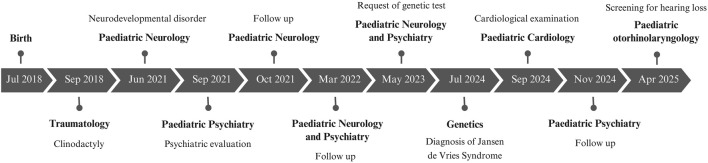
Timeline of patient diagnosis and follow-up.

Both siblings began behavioral therapy in Child Mental Health, receiving support from a psychologist and a speech therapist, as well as psychological support at school. Twin 1 has shown slower progress compared to Twin 2. The main milestones in their clinical history are presented in Figure 2.

### 2.2 Molecular diagnosis results

The twin brothers exhibited a karyotype with no structural or numerical changes (46XY). Furthermore, the copy number of the FMR1 gene CGG repeats, as determined by fragile X DNA testing, was found to be within the normal range. No pathogenic variations were detected in the copy number of the genes analysed by array-based comparative genomic hybridization with a 750 k custom array (CGH).

The exomes of both twins were sequenced and analysed, which confirmed that they are dizygotic (non-identical). The genetic analysis showed differences in their exomes, confirming that they are fraternal twins rather than identical.

WES Trio analysis of the proband revealed a previously undescribed heterozygous variant c.1411_1412del in the PPM1D gene (NM_003620.4) (Figure 1). This variant was classified as pathogenic (criteria PVS1, PS2 and PM2, Richards et al., 2015). This variant is a frameshift mutation that produces a premature STOP codon (p.Pro471Argfs*4), and is therefore predicted to cause loss of normal protein function by producing a truncated protein. The variant was confirmed by Sanger sequencing and the segregation study in the parents. Once paternity was confirmed, the segregation study showed that this pathogenic variant was *de novo*. Conversely, no pathogenic, likely pathogenic, or variants of uncertain significance were identified in the WES Trio analysed in his brother that could be associated with his clinical manifestations.

## 3 Methodology

The study was carried out in accordance with the Declaration of Helsinki of the World Medical Association and approved by the Local Ethics Committees. Informed consent was obtained from the family, after full explanation of the procedures.

### 3.1 M-CHAT™ - autism screening

The M-CHAT scale is comprised of 23 items (Robins et al., 2001). A notable advantage of the M-CHAT is its capacity for self-administration, thereby enabling parents to complete it at home or in the pediatrician’s waiting room. The scale is primarily designed for ages 16–30 months. The scale yields positive results when three of the 23 items or two of the key items are missing. The scale demonstrates a sensitivity of 87% and a specificity of 99% for the identification of ASD.

### 3.2 ADOS II module 2 questionnaire

The Autism Diagnostic Observation Schedule, Second Edition (ADOS-2) Module 2 is a standardized, semi-structured assessment tool designed to evaluate social communication, interaction, play, and restricted and repetitive behaviors in children aged 31 months and older who do not consistently use phrase speech (Lord et al., 2012).

### 3.3 Electroencephalogram

A 32-channel digital electroencephalogram (EEG) was recorded in accordance with standard office protocols using the 10–20 system with cap electrodes. Circular bipolar and double banana bipolar longitudinal montages were used for signal acquisition. The neurophysiological findings of the patients are outlined below: Wake EEG showed motion artefacts and baseline brain bioelectric activity characterised by theta frequency waves (5–7 Hz) in frontoparietal regions, interspersed with some beta frequencies. The occipital rhythm was observed at 6–7 Hz with a mean amplitude of 40–50 μV, maintaining stable synchrony and symmetry. No epileptiform paroxysmal abnormalities were detected, and no critical events were recorded during the session. The application of external light intermittent (ELI) stimulation did not result in the observation of significant alterations.

### 3.4 Neuroimaging study: acquisition and analysis

Data were acquired using an 8-channel head coil with a 3T system (Signa HDx, GE Medical System, Milwaukee, Wisconsin). Images were carefully inspected by an experienced neuroradiologist looking for structural defects; neuroimaging study and its analysis were performed before knowing the final clinical diagnosis and the result of the genetic study. DTI images were obtained using a SS-SE echoplanar Diffusion weighted image (DWI) sequence (TR:12000; FOV: 240 mm; sections thickness:3 mm, 0 spacing; matrix 128 × 128; bandwith: 250; 1 nex; diffusion encoding in 45 directions) with maximum b = 1,000 s/mm2. 3D-tractography was performed in an off-line workstation by using commercially available processing software as provided by the manufacturer (Functool 3D Fiber Tracking, GE, France) based on fiber assignment by contiguous tracking (FACT) method, achieved by connecting voxel to voxel. The threshold values were 0.3 for FA and 45° for the trajectory angles, between the regions of interest (ROIs). DTI tracts were also co-registered to the 3D-T1 weighted data set.

### 3.5 Conventional genetic studies

#### 3.5.1 Karyotype

Peripheral blood lymphocytes were cultured with phytohemagglutinin for 72 h, followed by cell harvest and conventional karyotyping. A total of 15 metaphases were examined, with a resolution of 400 GTG bands according to the presumptive diagnosis. A limitation of this study was that cryptogenic changes, monogenic or multifactorial conditions, low frequency mosaicism in other tissues, culture failure or contamination could not be excluded.

#### 3.5.2 Array CGH

A 750 k CYTOSCAN array from the Affymetrix commercial platform was used to hybridise the sample. This array was based on the simultaneous detection of CNV (copy number variation) probes and SNP (single nucleotide polymorphism) markers. For bioinformatic analysis, the manufacturer’s recommendations were followed and the minimum number of consecutive markers considered for SNP marker detection was 50. The minimum number of consecutive markers for CNV detection per gain and loss was 25 each. The average resolution achieved in intragenic regions (genes) was 1 marker. The average coverage was 1 marker per 1,737 bp, 1/6,145 bp and 1/4,125 bp for intragenic regions (genes), intergenic regions (nongene backbone) and total coverage (genes and nongene backbone), respectively.

#### 3.5.3 Analysis of the FMR1 gene CGG polymorphic region

The CGG polymorphic region in the promoter region of the FMR1 gene (NM_002024.5, Chr. X) was amplified by fluorescence PCR and TP-PCR. The amplified fragments were then analysed by capillary electrophoresis using an automated sequencer. According to the EMQN best practice guidelines for molecular genetic testing and reporting of Fragile X syndrome and related disorders (Biancalana et al., 2015), the reference ranges were as follows: normal allele (<45 CGG), intermediate allele (45–54 CGG), pre-mutation (55–200 CGG) and full mutation (>200 CGG). As a limitation, some rare cases caused by point mutations or deletions cannot be detected with this method.

### 3.6 Genomic study

The WES Trio procedure was performed on genomic DNA extracted from whole blood samples obtained from the proband, brother and parents. The genomic DNA was extracted from the blood samples using the Magna Pure 24 equipment (Roche Diagnostics). The quantity of extracted gDNA was measured with a fluorimeter (Quibit 3.0). Furthermore, the quality of the DNA obtained was determined by studying the absorbance ratios at 260/280 and 260/230 using the NanoDrop ND-2000 equipment. Furthermore, the integrity of the genomic DNA was analysed by means of electrophoresis in 0.8% agarose gels. Libraries were then prepared using the KAPA Hyper Plus Kit (Roche Diagnostics) in accordance with the manufacturer’s specifications and capture enrichment protocol with specific probes (KAPA HyperExome; Roche Diagnostics). Subsequent to this, we undertook massive parallel sequencing on a NextSeq550 instrument (Illumina). Subsequent signal processing, base calling, alignment, and variant calling were performed with Genologica variant analysis software (GenoSystem). This software, developed by Genologica, contains an optimised algorithm that includes the following steps: a) Initial quality control of the sequences, b) Filtering the sequences by eliminating indeterminacies, adapters and low quality areas, c) Second quality control of the sequences, d) Mapping on the Hg19 reference genome, e) Obtaining variants and CNVs, f) Mapping coverage study and g) Annotating variants. Finally, the variant prioritisation is based on stringent assessments at both the gene and variant levels, taking into consideration the patient’s phenotype and the associated inheritance pattern. The candidate variants were then visualised using IGV (Integrative Genomics Viewer). The evaluation of candidate variants was undertaken on the basis of rigorous assessments at both the gene and variant levels, with consideration given to the patient’s phenotype and the inheritance pattern. Variants were classified in accordance with the guidelines established by the American College of Medical Genetics and Genomics (ACMG) (Richards et al., 2015). A board of molecular clinical geneticists evaluated each variant classified as pathogenic, likely pathogenic, or a variant of uncertain significance. They then determined which, if any, should be reported. In each instance, the decision was taken to engage in a discussion with the referring physician and/or clinical geneticist regarding the potential causal variants.

## 4 Discussion

JDVS is a rare autosomal dominant disorder associated with truncating variants in exons 5 and 6 of the PPM1D gene. The clinical phenotype includes growth retardation, intellectual disability, distinctive facial features, small hands and feet, congenital malformations and various systemic complications. Despite the increasing number of reported cases, JDVS remains an underdiagnosed condition, partly due to the heterogeneity of its clinical presentation and the overlap of its symptoms with those of other neurodevelopmental disorders.

One of the major challenges in diagnosing JDVS is the significant phenotypic variability observed among affected individuals. Many symptoms, including global developmental delay, speech delay, behavioural abnormalities, and autistic features, are also present in other neurodevelopmental disorders, complicating clinical recognition. In the present study, twin brothers were both initially diagnosed with ASD, emphasising the challenges in differentiating JDVS from other conditions based solely on clinical presentation. While one of the twins carried a pathogenic PPM1D mutation, the other did not, yet exhibited notable behavioural differences. This case underscores the importance of molecular genetic testing in reaching an accurate diagnosis, as the clinical heterogeneity of JDVS can lead to misdiagnosis or delays in appropriate management.

In terms of clinical management, these patients require a comprehensive psychological evaluation and targeted therapy aimed at addressing their specific manifestations—in this case, language delay, social isolation, acquisition of normative behavioural patterns, sphincter control training, and frustration-tolerance techniques—delivered both in the clinic and within the school environment. The primary goal of such interventions is to foster greater autonomy and adaptive functioning.

WES analysis was conducted using a trio approach, including both parents, a methodological decision that ultimately proved to be pivotal in establishing the *de novo* nature of the pathogenic PPM1D variant. Conducting the analysis of the parents in a simultaneous manner not only confirmed the inheritance pattern but also facilitated the exclusion of inherited variants of uncertain significance, thereby enhancing the diagnostic accuracy. This approach is of particular importance in conditions such as JDVS, where phenotypic variability and incomplete penetrance can render clinical diagnosis unreliable. The findings of this study serve to reinforce the recommendation for comprehensive genetic testing, including trio-WES, in patients with neurodevelopmental disorders, especially in cases where clinical presentation does not fully align with known syndromes.

Additionally, cardiology and otolaryngology evaluations have been performed to rule out other complications. During clinical follow-up, improvements have been noted in both language skills and sleep patterns. However, some issues with sphincter control persist, and although behavioural problems have partially ameliorated, manipulative and defiant traits remain. School performance has shown a positive trajectory: Twin 2 has been discharged from Child Mental Health services, while Twin 1 continues with annual reviews.

Furthermore, the expanding clinical spectrum of JDVS, as demonstrated by recent case reports, suggests the need for further studies to refine genotype-phenotype correlations. The novel findings, including hepatomegaly, cleft lip and palate, vascular anomalies, and mosaicism-related variability, indicate that JDVS may have a broader spectrum of manifestations than previously recognised. Furthermore, the role of PPM1D in DNA damage response and tumour suppression gives rise to questions regarding potential long-term health implications, which necessitate further investigation.

In summary, this study underscores the diagnostic challenges associated with JDVS and the pivotal role of genetic testing, particularly trio-WES, in accurately identifying affected individuals. Early molecular diagnosis enables the implementation of tailored management strategies, enhanced genetic counselling, and a more comprehensive understanding of the condition’s entire clinical spectrum. Continued research is necessary to develop standardized diagnostic and therapeutic guidelines for JDVS as more cases are reported.

## Data Availability

The datasets presented in this study can be found in online repositories. The names of the repository/repositories and accession number(s) can be found in the article/Supplementary Material.
